# Structural analysis of herpes simplex virus by optical super-resolution imaging

**DOI:** 10.1038/ncomms6980

**Published:** 2015-01-22

**Authors:** Romain F. Laine, Anna Albecka, Sebastian van de Linde, Eric J. Rees, Colin M. Crump, Clemens F. Kaminski

**Affiliations:** 1 Department of Chemical Engineering and Biotechnology, Laser Analytics Group, Cambridge University, Pembroke Street, Cambridge, CB2 3RA UK; 2 Division of Virology, Department of Pathology, Cambridge University, Tennis Court Road, Cambridge, CB2 1QP UK; 3 Department of Biotechnology and Biophysics, Julius-Maximilians-University, Am Hubland, Würzburg, D-97074 Germany

**Keywords:** Super-resolution microscopy, Virus structures, Biophysics

## Abstract

Herpes simplex virus type-1 (HSV-1) is one of the most widespread pathogens among humans. Although the structure of HSV-1 has been extensively investigated, the precise organization of tegument and envelope proteins remains elusive. Here we use super-resolution imaging by direct stochastic optical reconstruction microscopy (*d*STORM) in combination with a model-based analysis of single-molecule localization data, to determine the position of protein layers within virus particles. We resolve different protein layers within individual HSV-1 particles using multi-colour *d*STORM imaging and discriminate envelope-anchored glycoproteins from tegument proteins, both in purified virions and in virions present in infected cells. Precise characterization of HSV-1 structure was achieved by particle averaging of purified viruses and model-based analysis of the radial distribution of the tegument proteins VP16, VP1/2 and pUL37, and envelope protein gD. From this data, we propose a model of the protein organization inside the tegument.

**Supplementary information:**

The online version of this article (doi:10.1038/ncomms6980) contains supplementary material, which is available to authorized users.

## Abstract (web summary)

Herpes simplex virus type-1 (HSV-1) is a widespread human pathogen, the structure of which is not yet fully characterized. Here, the authors apply *d*STORM super-resolution microscopy in combination with advanced data analysis tools to locate the position of four key protein layers in HSV-1 with unprecedented precision.

**Supplementary information:**

The online version of this article (doi:10.1038/ncomms6980) contains supplementary material, which is available to authorized users.

## Graphical abstract

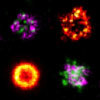

## Introduction

The *Herpesviridae* comprise a large family of enveloped DNA viruses that infect a wide range of hosts, including humans. One of the most widespread human members of this family, herpes simplex virus type-1 (HSV-1), is a common cause of cold sores but can also result in herpes keratitis^1^, a leading infectious cause of blindness, and herpes simplex encephalitis^2^, which has a high mortality even in patients treated with antiviral therapy. It is highly contagious and can be easily passed from individual to individual via direct contact. In addition, after primary infection, HSV-1 establishes a lifelong latent infection in sensory ganglia with periodic reactivation of the virus leading to both symptomatic and asymptomatic virus shedding^3^.

Electron microscopy (EM) and electron tomography (ET) studies of HSV-1 have revealed that virus particles have a spherical shape^4^, with diameter ranging from 155 to 240 nm, each containing an icosahedral capsid with a diameter of 125 nm^5,6^, which contains the viral DNA. The structure of the highly ordered capsid complex has been studied extensively and is now well defined. A partially ordered layer of tegument proteins surrounds the nucleocapsid and the viral envelope encloses the nucleocapsid/tegument core. The envelope carries a set of glycoproteins that are essential for virus entry and viral morphogenesis. Among them, glycoprotein D (gD) mediates interactions with cellular receptors and initiates the cascade of events essential for HSV-1 entry into target cells^7^. The tegument is the most structurally complex layer of the virus, and so far, over 20 tegument proteins have been identified for HSV-1 (ref. 8). In particular, VP1/2 (also known as pUL36) is the largest tegument protein (>330 kDa) and is essential for HSV-1 entry and assembly^9,10^. Owing to the interaction of its carboxy terminus with the minor capsid protein pUL25, VP1/2 is often referred to as being a part of the inner tegument. With its binding partner pUL37 (refs 11, 12), VP1/2 plays an essential role in capsid transport on microtubules^13^. In contrast to VP1/2 and pUL37, one of the major tegument proteins VP16 is thought to reside closer to the viral envelope and be a part of the outer tegument. VP16 also interacts with VP1/2 (ref. 14) and induces transcription of immediate-early viral genes, thus playing an important role in the viral life cycle^15^. Owing to its multiple interactions with other viral proteins, VP16 has been proposed to serve as a central organizer for the tegument^12^.

Although it is now clear that the tegument is not as disordered as previously thought, its structure and organization are still poorly understood. Structural information is also missing on the distribution of specific glycoproteins in the virus envelope. EM studies of biological materials are often restricted to highly ordered structures and, therefore, cannot be used to resolve viral tegument and envelope organization. Moreover, although EM and ET are powerful tools to visualize overall physical structure, their ability to localize specific proteins is limited. Optical techniques do not suffer this latter disadvantage; however, they are limited by optical diffraction, with a typical lateral resolution of ~200 nm. This resolution is comparable to the entire virus diameter and thus too coarse to reveal structural information. Recently, a host of sub-wavelength resolution optical imaging methods have been developed to circumvent the diffraction limit^16,17,18^. These super-resolution methods include stimulated-emission depletion microscopy^19^ and single-molecule localization microscopy (SMLM), such as photoactivated localization microscopy (PALM)^20^, stochastic optical reconstruction microscopy^21^ (STORM) and direct STORM (*d*STORM)^22^. Although PALM^20,23^, stimulated-emission depletion^24^ or *d*STORM^25,26,27^ have begun to be used in the investigation of viral replication and structure, their application in the context of virology research is still in its infancy.

In particular, the lateral resolution achievable with SMLM (typically 20–30 nm, full width at half maximum) is well suited for the study of HSV-1 particles. The increased resolution in SMLM is based on the temporal separation and the subsequent precise localization of individual fluorescent emitters. Compared with other super-resolution methods, not only can the fluorophore localizations be used for the reconstruction of a high-resolution image, but they also allow for the counting of molecule numbers^28,29,30^, cluster analysis^31,32^ or geometric modelling^33^.

Here we present a study of HSV-1 viral structure using *d*STORM. First, multi-colour localization microscopy was used to visually distinguish virus proteins in individual virus particles. We then designed a model-based analysis of SMLM data and used particle averaging (a method routinely used in EM^34^) to reconstruct a high-resolution image and to determine the position of individual protein layers within the virus particle with nanometre precision. We additionally investigated the effect of the fluorophore linker size and compared immuno-labelling strategies in the context of model-based analysis. Finally, our data set confirmed and quantified the spatial offset of the capsid with respect to the centre of the virion.

## Results

### Multi-colour *d*STORM resolves protein layers in HSV-1

**Figure 1:**
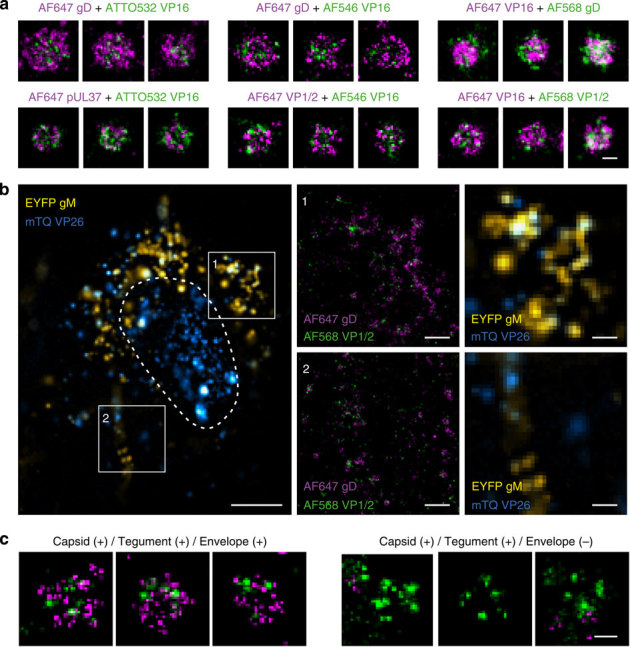
Multi-colour *d*STORM analysis of purified and cellular HSV-1 particles. (**a**) Representative two-colour *d*STORM images of purified virus particles obtained from various combinations of co-labelling, showing the relative position of protein layers within individual virus particles. Top panel shows labelling of the envelope and the tegument, whereas bottom panel presents labelling of two tegument proteins. (**b**) Four-colour imaging of HSV-1 in a fixed HFF-TERT cell. The nucleus is indicated by a dashed line. Cells were infected with VP26-mTurquoise/gM-EYFP recombinant virus for 12 h and then immunostained against gD (AF647) and VP1/2 (AF568), using secondary labelling. In the two insets (1, 2) showing zoom-ins, the two super-resolved images are overlaid (AF568 VP1/2 and AF647 gD, green and magenta, respectively) and the mTurquoise (mTQ) and EYFP channels are displayed. (**c**) Representative capsid-positive virus particles observed in cells showing the presence or absence of envelope. Scale bars, 100 nm (**a**,**c**), 5 μm (**b**), 1 μm (**b**1, **b**2).

We used two-colour *d*STORM imaging of purified virions to investigate the architecture of different protein layers within individual virus particles. The use of Alexa Fluor (AF) 647 is well established in localization microscopy, owing to its favourable photoswitching behaviour and its high photostability and quantum yield. We thus searched for a second fluorophore that could be reliably used in combination with AF647 for imaging virus particles and tested AF647/ATTO532, AF647/AF546 and AF647/AF568 in a range of photoswitching environments. All fluorophores were linked to secondary antibodies and imaging protocols were optimized for each fluorophore combination (Fig. 1a).

As shown in the top row in Fig. 1a, for every fluorophore combination tested, two-colour *d*STORM consistently demonstrated the envelope protein gD to be located at larger radial distance than the tegument protein VP16. The two tegument proteins VP16 and pUL37 showed a large overlap and similar radial distributions, suggesting that VP16 and pUL37 reside in the same region of the tegument layer (Fig. 1a, bottom row). The inner tegument protein VP1/2 and the outer tegument protein VP16 are thought to reside at different radial locations, with VP1/2 expected to lie closer to the capsid than VP16. However, by resolving and analysing individual virus particles, we could not consistently differentiate between outer and inner tegument, irrespective of dye combination used (Fig. 1a, bottom row, second and third panel).

### Two-colour *d*STORM resolves virus particles in infected cells

Next, we imaged virus particles within infected cells by using two-colour *d*STORM. Here, to aid identification of the virus assembly state, we produced a fluorescently tagged recombinant HSV-1 that genetically encoded the small capsid protein VP26 fused to mTurquoise and the envelope protein gM fused to enhanced yellow fluorescent protein (EYFP). We were therefore able to locate individual capsids and discriminate between enveloped and non-enveloped particles within the infected cells. AF647 and AF568 were then used to label envelope protein gD and tegument protein VP1/2, respectively. The capsid protein VP26 (shown in blue in Fig. 1b) was primarily present in the nucleus (where capsids are assembled) and, less abundantly, in the cytoplasm where the punctate pattern indicates the presence of individual capsids. The envelope protein gM (shown in yellow) was mostly present in the perinuclear region of the cytoplasm and near the plasma membrane, as expected for a viral envelope glycoprotein that undergoes vesicle-based transport through the secretory and endocytic compartments of the cell^35^. *d*STORM imaging of both AF647 and AF568 allowed the identification and characterization of structural elements of individual particles (tegument and envelope) directly in the infected cells and the mTurquoise fluorescence image was used to identify the particles that were capsid positive. Among the capsid-positive particles, two main types were observed: those containing both tegument and envelope (Capsid(+)/Tegument(+)/Envelope(+)) and others that were devoid of envelope (Capsid(+)/Tegument(+)/Envelope(−)) (Fig. 1c). The particles exhibiting all viral components (Capsid (+)/Tegument(+)/Envelope(+)) displayed structures that were consistent with those observed in purified viruses (Fig. 1a). In particular, VP1/2 (tegument) consistently appeared to be contained within the gD layer (envelope). We observed no clear visual difference in the distribution of VP1/2 between virus particles that were envelope positive and envelope negative.

These data demonstrate that the use of two-colour *d*STORM in combination with two-colour wide-field fluorescence (using genetically expressed mTurquoise-VP26 and gM-EYFP) provides super-resolution information about individual virions at different assembly steps.

However, we noted that the mean localization precision (defined further on as 1 s.d.) achieved for the two-colour *d*STORM images obtained both in cells and in purified viruses was of the order of 10–15 nm, irrespective of the fluorophore and the antibody used. We also observed that the fraction of localization with high-localization precision (5–10 nm) was much greater for AF647 than AF568, AF546 or ATTO532. This observation is in agreement with AF647 being one of the brightest fluorophore commonly used for *d*STORM.

### Particle averaging and model-based analysis of HSV-1

Nanometre-scale structural analysis by SMLM is a developing field. However, it requires high spatial resolution and high labelling specificity, and typically involves complex data analysis. This complexity is notably due to the need for assessing effects of the localization error and the linker size between the protein of interest and the fluorescent label^36,37^. Here we present a structural analysis method taking these effects into account and apply it for the precise determination of the distribution of important tegument and envelope proteins in HSV-1.

**Figure 2:**
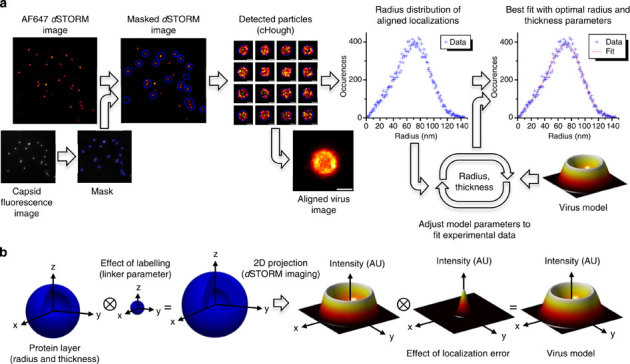
Virus particle alignment and model-based analysis. (**a**) Flowchart representing the main data analysis steps. The image analysis procedure was performed using a custom-written MATLAB routine. Briefly, the *d*STORM image was masked using the mTurquoise and AF568 fluorescence image, and the circular Hough (cHough) transform was applied to detect the particles and determine their centres. The radial distribution of localization is obtained from the aligned localizations and then fitted to the MCV model. (**b**) MCV model. The effect of the labelling linker, the imaging (projection) and the localization error are taken into account. The ⊗ symbol represents the convolution operation (for more details, see Supplementary Notes 1–3).

We acquired single-colour *d*STORM images of purified viruses labelled with AF647. Here, a recombinant virus expressing mTurquoise-VP26 was used. We imaged a large number (>50) of particles and data sets were analysed as shown in Fig. 2a (see also Supplementary Notes 1–3 and Supplementary Figs 1–3). We ensured that only fully assembled virus particles, for example, containing capsid, tegument and envelope, were used for the analysis. For this, capsid-positive particles were selected by creating a mask from the mTurquoise-VP26 fluorescence image. A second mask was also created from an additional wide-field fluorescence image using AF568 as described in the Methods section.

**Figure 3:**
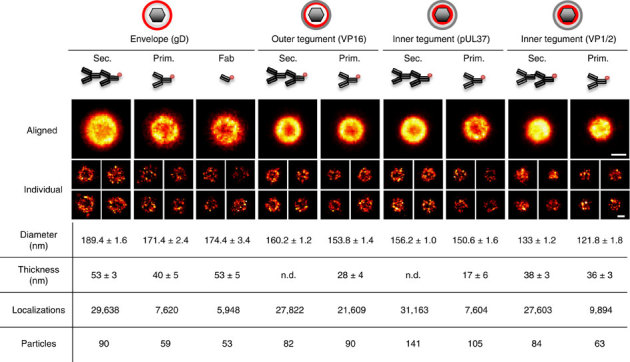
Aligned particle images and results from the model-based analysis. Virus images obtained from aligned particles (aligned) and representative individual particles (individual). In the table, results from the model-based analysis are shown. The total number of localizations and number of particles are also indicated. The errors shown in the table are the 95% confidence interval obtained from the fit. n.d., not determinable. The radial distribution and the optimal fit are shown in Supplementary Fig. 3. Scale bar, 100 nm.

Images of individual virus particles (as those shown in Figs 2a and 3) were typically reconstructed from 100 to 300 localizations, with a typical localization precision of 5–10 nm (1 s.d. as described by Mortensen *et al.*^38^, see Supplementary Note 3). By aligning the obtained particles, the localizations can be combined to reconstruct a high-density super-resolution image of a specific protein layer. Furthermore, with the large number of localizations obtained in the aligned data set, the radial distribution of the aligned localizations can be accurately retrieved and analysed to determine the position of this individual layer. For this, the radial distribution of localizations was fitted to a Monte-Carlo model of virus localization data set (Monte-Carlo-based virus (MCV) model, presented in Fig. 2b and Supplementary Note 2), developed assuming spherically symmetric virus particles. In this model, each viral protein is assumed to lie within a spherical shell described by two parameters: the shell diameter and the shell thickness. The model takes both the linker size and the localization error into account. The shell diameter and thickness are the structural parameters of interest and are both obtained by fitting, whereas the linker size and localization precision are first estimated and then fixed during the analysis (Supplementary Note 3). The shell diameter represents the average radial position of the protein of interest in a population of virus particles. On the other hand, the shell thickness obtained by our method is a result of several contributions: the actual thickness of the protein layer (the thickness of the shell shown in Fig. 2b), the variability of the diameter from particle to particle in a population of virus particles and any deviation from the spherical symmetry.

First, we investigated the effect of the fixed parameters (linker size and localization precision) on the fitted parameters. The estimation of the shell diameter was found to be very robust with respect to the fixed parameters and errors of 50% in linker size or localization error estimates only introduced a bias of <5%. However, the shell thickness is more strongly affected by such errors. An overestimation of either linker length or localization precision leads to an underestimation of the shell thickness (and vice versa). Details can be found in Supplementary Note 4 and Supplementary Fig. 4.

The analysis presented here allows the quantification of both diameter and thickness of individual protein layers from localization data sets and provides much greater precision than single-particle imaging.

### Direct labelling of viral proteins

To assess the effect of linker size, we compared three different antibody-based labelling methods: indirect immunochemistry (successive primary and secondary antibody labelling, termed secondary labelling), direct dye-conjugated primary antibody (primary labelling) and direct dye-conjugated Fab fragments (Fab labelling), all conjugated with AF647. We estimated the corresponding linker sizes as 20, 10 and 5 nm, respectively, based on the structure of the IgG (PDB 1IGT^39^). The analysis was performed for the envelope protein gD and the three tegument proteins VP16, pUL37 and VP1/2.

In general, Fab fragments are preferable for super-resolution microscopy due to their small size. However, the specificity of the Fab fragments we produced was often compromised and efficient labelling was only achieved using the anti-gD Fab fragment. This is likely to be due to antibody structure and/or dye conjugation within the antigen-binding region, leading to lower affinity or avidity interactions between the specific epitopes. Therefore, only results from anti-gD Fab fragment are shown. Figure 3 shows the image of the virus obtained from particle alignment as well as representative single-particle images obtained from the raw *d*STORM images for each protein and labelling combination. The total number of localizations, the number of particles and the results from the MCV model fits are stated in the table. Radial distributions and the optimal fits for all data sets are shown in Supplementary Fig. 3.

Irrespective of the labelling approach, the envelope protein gD exhibited the largest diameter, VP16 and pUL37 showed a diameter consistent with their tegument location (between capsid and envelope), whereas the inner tegument protein VP1/2 appeared very close to the capsid (from the known capsid diameter of 125 nm^5,6,40^).

The reduction in the number of localizations that was observed when comparing the secondary with primary and Fab labelling strategies can be attributed to the reduction of the average number of fluorescent labels per antibody, from 3 to 4 on commercial secondary antibody to 1.0–1.5 for in-house labelled primary antibodies and Fab fragments. In addition, multiple secondary antibodies may bind to a single primary antibody. This would also contribute to the greater number of localizations observed with secondary labelling.

As our analysis takes into account the linker size, different labelling strategies should lead to the same results. Any differences can then be interpreted as deviations from the model (in particular, non-spherical or non-random distribution) or direct effect of the labelling on the particles (labelling affecting the particle size, for instance). In fact, we observe that for VP16, pUL37 and VP1/2, changing the labelling from secondary to primary resulted in a small reduction of the diameter (by 5–10 nm). This small reduction may be a result of the heavy loading of the tegument with large labels that occurs with secondary labelling, leading to an observed swelling of the particle. The compact and rigid structure of the capsid may also contribute towards pushing the labels outwards rather than inwards.

For gD, the reduction in diameter from secondary to primary labelling is more pronounced than for the tegument proteins (~20 nm as opposed to 5–10 nm). In this case, the difference can be explained by a deviation between the envelope protein structure and the model. Instead of being randomly distributed around the average radial position of the protein layer, the labels may have a tendency to stretch outwards, away from the centre of the particle, and therefore smaller labels admit a smaller bias. This results in an overestimation of the protein diameter due to the linker size, which is then more pronounced in the case of the secondary labelling. An additional ~20 nm on the diameter corresponds to ~10 nm on the radius, which is in agreement with the size of a single antibody. No significant reduction in the diameter of the protein layer was observed between the primary and the Fab labelling, suggesting that the precision of our measurements is not limited by the size of the labelling once the distance between the protein of interest and the fluorescent label is of the order of or below the size of a single complete antibody.

The measurements of thickness obtained for gD are consistent across the three different labelling strategies. Similarly, the thicknesses recovered by primary and secondary labelling for VP1/2 are in good agreement with each other. We note that for VP16 and pUL37, the use of secondary labelling did not allow the estimation of the thickness parameter. This highlights that the use of primary labelling allows the measurement of smaller thickness that secondary labelling cannot reliably determine. A thorough analysis of the effect of the linker size (Supplementary Note 5) showed that the shell thickness may appear non-determinable if the estimation of the linker size is incorrect. This analysis also demonstrated that the linker size for the secondary labelling is more variable (between 15 and 25 nm for this data set depending, on the protein labelled) than for primary labelling. This variability in linker size using the same labelling approach is very likely to introduce bias or non-determinable thickness when the linker cannot be estimated precisely. On the other hand, a linker size of 10 nm for primary labelling provided reliable and consistent results.

As the secondary labelling is more subject to biases for model-based analyses, we conclude that the primary labelling appears the most robust method to measure the diameter and thickness of a discrete protein layer and is therefore the most appropriate approach for *d*STORM structural analysis.

### Modelling HSV-1 protein layer architecture

**Figure 4:**
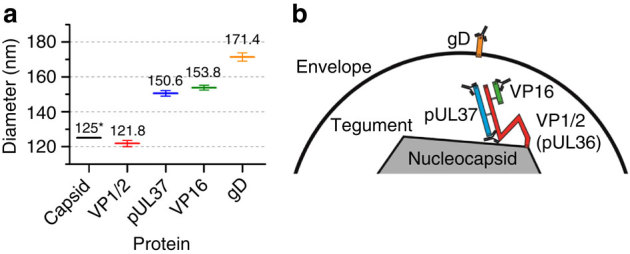
Model of protein distribution inside the tegument of HSV-1. (**a**) Average diameter of protein layers, error bars are 95% confidence interval obtained from the fit. (**b**) HSV-1 protein architecture model. This model was built from the diameters obtained using primary labelling and biochemical evidences of interaction sites and antibody-binding sites. *The diameter shown for the capsid was obtained from published EM data^5^.

From the model-based analysis using primary labelling, the diameter of different protein layers could be obtained with high precision (1–2 nm error, 95% confidence interval). As shown in Fig. 4a, the diameter obtained decreased from envelope (171.4±2.4 nm) to the tegument proteins VP16 (153.8±1.4 nm), pUL37 (150.6±1.6 nm) and VP1/2 (121.8±1.8 nm). Moreover, the distance between the capsid and the envelope (23±4 nm) can be interpreted as a measurement of the average tegument thickness.

The diameter obtained here for VP16 is in good agreement with the observation that it interacts with a number of envelope-anchored proteins^41,42^ and therefore is expected to localize close to the envelope. For VP1/2, it has been shown that the C-terminus of VP1/2 interacts with the minor capsid protein pUL25 (ref. 43), but the structure and the spatial arrangement of the remaining part of this large protein remains elusive. Here we were able to show that the binding site for the VP1/2-specific antibody used here (located between the amino acids 1564 and 1876) resides very close to the edge of the capsid. Furthermore, despite the common view of pUL37 as an inner tegument protein (notably because it interacts with VP1/2 (ref. 12)), the data we obtained here provide evidence that pUL37 is located at a similar position in the tegument to VP16 and, hence, closer to the outer part of the virion than previously thought.

From our measurements, we propose a model for HSV-1 tegument architecture (Fig. 4b). In addition, this model includes the current understanding of the interaction and antibody-binding sites of the considered proteins in the tegument. Indeed, we have previously shown that VP16 binds to VP1/2 within the first 361 amino acids of VP1/2 (ref. 14). Moreover, the binding site of VP1/2 to pUL37 has been localized within the C-terminal region of pUL37 (ref. 11). The diameter measured for pUL37 (153.8 nm) is in good agreement with published data showing that the interaction site of VP1/2 with pUL37 is close to the VP16 interaction domain, within the amino terminus of VP1/2 (ref. 44), potentially placing both VP16 and pUL37 in a similar location relative to VP1/2.

Although interpretation is not as straightforward as for the shell diameter data, the shell thickness also provides structural information. For instance, as the gD protein layer is part of the membrane structure (with no intrinsic thickness), the large thickness obtained for gD (40 nm) indicates a large variability of the envelope diameter from particle to particle (supporting the large range of particle sizes found by EM) and/or a potential deviation from the spherical symmetry, underlying the plasticity of the envelope. On the other hand, the thickness of VP16 and pUL37 appear smaller than that of gD, which is consistent with the notion that there is much less plasticity in the virus tegument compared with the envelope layer. At the moment, we do not have a clear explanation as to the observed differences in the thickness for VP1/2 and pUL37. Reasons could include differential labelling efficiencies for the two proteins and dense packing of fluorophores on the small shell of VP1/2, increasing localization artefacts. An underestimation of the alignment error would also lead to an overestimation of the shell thickness (see Supplementary Note 4). Finally, there may be differential ‘plasticity’ of the two protein layers, leading to a real geometric distortion.

### Spatial offset of the capsid

Cryo-ET studies indicated that HSV-1 particles are not symmetrical^4,45^. These studies demonstrated that the capsid does not lie in the centre of the particle. This spatial offset may be explained by the structure and function of the portal vertex, which is thought to form a connection between the capsid and the envelope^45^. A spatial offset between the centre of the capsid and the outer protein layer of ~15 nm was measured by ET^4^.

Here we developed a method to estimate the average distance between the centre of the capsid and the centre of the outer protein layer (tegument or envelope), as detailed in Supplementary Note 6. Briefly, the displacement between the centre of the considered layer (tegument or envelope, determined by a fitted circle) and the centre of the capsid (determined by localization of the centroid of the mTurquoise-VP26 fluorescence) was calculated for a large number of particles and the data were analysed with a further model-based analysis. This approach is related in nature to that of a recent investigation of sub-virion protein distribution in the related herpesvirus pseudorabies virus^46^. In the latter work, the displacement between the centroids of the wide-field fluorescence patterns from dually labelled virions was determined with high precision. The results of our analysis are shown in Supplementary Fig. 5b. After correction for the residual chromatic random error, the offset was estimated as 12, 7 and 24 nm for gD, VP16 and pUL37, respectively. The order of magnitude of this offset is in good agreement with the value determined by ET^4^, although greater than expected for pUL37.

## Discussion

Traditionally, fluorescence-based studies of HSV-1 structure have been limited by the small size of the viral particles. Here we used *d*STORM super-resolution microscopy to investigate and quantify the organization of key proteins within HSV-1 virions. Our results provide intriguing details on tegument architecture in mature HSV-1 virions that are not accessible to traditional EM or ET techniques. Our work establishes *d*STORM as a powerful new tool in deciphering virus structure.

First, we conjugated a number of antibodies with *d*STORM-compatible organic dyes and optimized labelling protocols to minimize background from nonspecific antibody binding. With two-colour *d*STORM, we were able to visualize and discriminate between tegument and envelope protein layers within single virus particles. The organization of important tegument and envelope proteins could then be observed both in purified viruses and in infected cells. The method thus shows great potential as a structural tool to study the organization and interaction of viral and cellular proteins during various stages of virus replication.

Structural information of biological nanostructures can be derived through averaging repetitive features as is commonly performed in EM. Particle averaging in SMLM images was originally shown for the nuclear pore complex^34,47^, and more recently for HIV-1 (ref. 27). Here we extended this approach and developed a model-based analysis of SMLM data to extract structural parameters for specific virus proteins layers with optimal precision (1–2 nm for the shell diameter and <6 nm for the thickness). We compared different antibody-labelling methods in the context of structural analysis and found that primary labelling offers the most robust readout of protein location. We found that errors in the estimates of linker size or localization error introduce bias in the analysis and this needs to be carefully considered when secondary labelling is used: for example, we found that linker lengths varied from *ca*. 15 to 25 nm depending on which protein was labelled; therefore, precise estimates may be difficult in a practical situation. In contrast, we found that primary labelling resulted in a reliable and constant linker length of 10 nm. Fab fragments offer potential for even shorter linker size. However, producing Fab fragments that retained sufficient binding specificity and/or affinity proved challenging for most of the proteins we considered. In future, other labelling techniques may offer more promising routes towards short linker size, for example, nanobodies^48^ or aptamers^49^. Furthermore, the effect of restricted rotational freedom is of great importance, as it affects the localization precision^50^. We show that for analysing purified virions, primary labelling offers a good compromise, introducing small to no bias and providing high localization precision.

We used our model-based analysis to accurately determine the diameters of different protein layers in purified virions and were thus able to build a structural model of the HSV-1 tegument architecture (Fig. 4). We observed significant differences in the diameter of all investigated protein layers and found that the central section of VP1/2 is located deep within the virus particle, featuring a diameter of only ~122 nm. This is similar in size to the capsid itself, as established by prior EM studies^5^. This observation suggests that the central domain of VP1/2 may directly interact with the capsid, similar to the C-terminus of VP1/2 (ref. 43). In addition, our model suggests that the N-terminus of VP1/2 projects away from the capsid towards the envelope, as both pUL37 and VP16 interact with the N-terminal domain of VP1/2 and were located near the envelope (Fig. 4b). To investigate this further, it would be interesting to measure particle diameters using antibodies specific to various epitopes along the length of VP1/2, in particular the N-terminus.

In addition, our data demonstrate that the spatial distribution of tegument proteins within a virion cannot necessarily be inferred from the stability of their attachment to the capsid. pUL37 is commonly defined as an inner tegument protein, partly due to its interaction with VP1/2 (ref. 12) and the resistance of both these proteins to removal from purified capsids^51^. Here we have shown that the N-terminal region of pUL37 is located close to VP16 and in proximity to the envelope. Interestingly, pUL37 has recently been shown to interact with the gK/pUL20 envelope protein complex^52^, implying that pUL37 could span the tegument, linking VP1/2 to the envelope. In addition, VP16 is known to interact with the cytoplasmic domains of other envelope glycoproteins^41,42^. These previous findings support our observations using super-resolution microscopy, which showed that both VP16 and pUL37 are located close to the envelope. It will be important in the future to also determine the spatial distribution of other tegument proteins to complete the model of the architecture of this complex structural domain.

It would be interesting to take advantage of the molecular counting capabilities of SMLM techniques, as described for PALM^28,29^ via the use of photo-activatable fluorescent protein and more recently for *d*STORM^30^ via immunolabelling with synthetic dyes. However, this approach requires extensive calibration protocols^30^ and will be the topic of future studies.

We were also able to resolve and quantify the capsid spatial offset in HSV-1 particles, using precise localization of both the centre of the capsid and those of the other protein layers (tegument or envelope). We observed offsets in good agreement with data previously obtained by cryo-ET^45^. Our method is based on the superposition of two-dimensional (2D) *d*STORM data obtained from several particles, and the analysis of radial distributions of the offsets taking the three-dimensional (3D) structure of the particle into account. The spatial offset between the capsid and several tegument proteins in a related alphaherpesvirus, pseudorabies virus, has also been reported in an elegant study by Bohannon *et al.*^46^ Our analysis expands on this approach, which uses conventional fluorescence imaging, through the application of super-resolution microscopy. Super-resolution microscopy provides detailed images of individual virus particles, and so it is possible to determine their centre with greater confidence and accuracy. For example, non-uniformity in the fluorescence signal that may arise from incomplete labelling, or geometric defects, are directly observable by SMLM but are hidden when using diffraction-limited imaging techniques. Although we did use conventional fluorescence data for the determination of the capsid centre, the high copy number of VP26 and the rigid structure of the capsid ensure that an isotropic signal is obtained from mTurquoise; therefore, the centroid of the ensemble localization provides an accurate determination of the capsid centre. Surprisingly, our measurements of capsid offset relative to pUL37 gave a larger value (24 nm) than relative to VP16 (7 nm), which is difficult to reconcile with the notion that these two tegument proteins are both co-ordinated by interaction with a similar region of VP1/2. However, it is well established that there is a significantly greater number of VP16 proteins per virion (>600) than either pUL37 or VP1/2 (both <200)^53^. Furthermore, VP16 interacts with several other tegument and envelope proteins in addition to VP1/2 (ref. 54). As the structure and orientation of pUL37 and VP16 within the tegument have yet to be determined, whether bound to VP1/2 or not, it is unclear whether this discrepancy in spatial offset for VP16 and pUL37 is a genuine reflection of a different intra-virion distribution of the proteins or not. Future studies with other specific antibodies against VP16 and pUL37 should clarify this.

We have demonstrated the use of 2D *d*STORM and particle averaging for the precise determination of 3D protein radial distribution in virus particles. In principle, 3D SMLM imaging techniques can also be used, such as *d*STORM with astigmatic^55^ or biplanar^56^ detection schemes. Our method retain the high lateral resolution of 2D *d*STORM techniques while recovering 3D information by taking into account the spherical symmetry of the particle. The same approach is applicable for any spherical system, such as other complex viruses, including coronaviruses (for example, the SARS, severe acute respiratory syndrome, or MERS, Middle East respiratory syndrome, coronaviruses) and rotaviruses. However, the spherical symmetry is not an *a priori* constraint: any system is amenable to the methods presented here as long as structural information is available for accurate modelling of the SMLM data. For more complex structures, the alignment may require more complex algorithms, such as those reported for the alignment of nuclear pore complexes^34^. Another powerful approach to consider would be correlative imaging, combining optical fluorescence microscopy and EM as demonstrated previously^57^. This approach would potentially reveal structural information (notably on the capsid) to complement the molecule-specific fluorescence imaging.

In summary, we have developed the use of *d*STORM combined with particle averaging and modelling, to reveal hitherto unknown structural details of a complex virus. The data obtained with the presented methods establish SMLM as a high precision tool for structural investigations of virus particle architecture, offering excellent molecular specificity and the potential for the use in complex biological environments.

## Methods

### Cell lines and viruses

HaCaT cells^58^ were grown in Glasgow minimal essential medium supplemented with 10% FCS, 2 mM glutamine, 100 U ml^−1^ penicillin and 100 g ml^−1^ streptomycin. Vero cells (from ATCC) and telomerase immortalized human foreskin fibroblasts (HFF-TERT^59^) were maintained in DMEM completed with 10% fetal bovine serum (FBS), 2 mM glutamine, 100 U ml^−1^ penicillin and 100 g ml^−1^ streptomycin. A bacterial artificial chromosome cloned HSV-1 genome isolate (strain KOS) was used in these studies and was described previously^60^. VP26-mTurquoise and EYFP-gM viruses were prepared using Red Recombination of bacterial artificial chromosome cloned HSV-1 genome^61^ by replacing amino acids 1–4 of VP26 with mTurquoise and adding EYFP(A206K) to the C-terminus of gM. To prepare purified viruses, HaCaT cells were infected at 0.01 plaque-forming unit (PFU) per cell and incubated for 3 days. Supernatants were collected and cleared by low-speed centrifugation. The viruses obtained from the culture supernatants were pelleted at 24,000 *g* for 2 h and resuspended in PBS with 1% FBS. Viruses were further purified by 5–15% Ficoll gradient ultracentrifugation at 17,500 *g* for 1.5 h. Visible virus band was collected and spun down at 49,000 *g* for 2 h. Pellet was resuspended in PBS, aliquoted and stored at −70 °C. Virus titre was assessed by titration in Vero cells.

### Antibodies preparation and labelling

Monoclonal antibodies against VP16 (LP1; Abcam (Ab110226)), gD (LP2) and VP1/2 (CB4) were described previously^14,62,63^. To generate HSV-1 pUL37-specific monoclonal antibodies, female BALB/c mice were infected with HSV-1 (strain 17) by ear scarification followed by an intraperitoneal boost 1 month later. Spleens were harvested 3 days later, and B-cell hybridomas were generated as previously described^64^. Hybridoma supernatants were screened for reactivity in immunofluorescence assays by using cells transfected with an HSV-1 pUL37 expression plasmid. A cloned hybridoma line secreting antibodies with a strong reactivity to HSV-1 pUL37 was selected and named CB8. Secondary AF antibodies were purchased from Molecular Probes, anti-mouse IgG1 conjugated to ATTO 532 were from Rockland antibodies and assays. AF647 succinimidyl ester was from Molecular Probes. Monoclonal antibodies were purified from hybridoma supernatants using Protein A or G sepharose 4B fast flow from Sigma. Fab fragments were prepared using Pierce Mouse IgG1 Fab and F(ab′)_2_ preparation kit for LP1 (IgG1) and Pierce Fab Preparation Kit for LP2 (IgG2a), CB8 (IgG2a) and CB4 (IgG2b), following the manufacturer’s instructions. Primary antibodies and Fab fragments were labelled with succinimidyl ester AF probe as follows. Probe was dissolved in dissolved in dimethylformamide to concentration 1 mg ml^−1^ immediately prior reaction. Molecular ratio of probe to protein was set to 2.5. One hundred micrograms of antibody/Fab was incubated with probe in 50 mM NaHCO_3_ for 1 h. Labelled antibody was purified from unbound dye directly after reaction using NAP5 Sephadex G-25 column from GE Healthcare. Average degree of labelling was between 1.0 and 1.5, as determined using instructions from Molecular Probes.

### Labelling viruses

Lab-Tek II chambers were first blocked with 2 M glycine and coated with poly-L-lysine. Viruses (4 × 10^6^ PFU) was bound for 1 h and then fixed with 3% EM-grade formaldehyde. Blocking and permeabilization was performed with 0.1% Triton X-100, 2% FBS in PBS and followed by antibody binding. Typically, primary antibodies were used at 100 μg ml^−1^ and secondary at a dilution of 1/500. An additional post-staining fixation step using 3% EM-grad formaldehyde was included to prevent antibodies detaching from antigens.

### Infection assay

HFF cells were seeded onto Lab-Tek II dish after glycine blocking the plate. The next day, cells were infected with KOS VP26-mTQ/gM-EYFP virus at 5 PFU per cell. After 12-h infection, cells were fixed, permeabilized and labelled with antibodies as described above.

### *d*STORM imaging

The imaging was performed on inverted total internal reflection fluorescence (TIRF) microscopes (Nikon TE-300 and Olympus IX-71, Japan) custom-built for *d*STORM acquisition, as previously described^22,65,66^. The Nikon TE-300 microscope was equipped with an iXon3 897 EM-CCD camera (Andor, UK) and the Olympus IX-71 was equipped with two iXon DU897 EM-CCD cameras (Andor). The dyes were irradiated with lasers and their fluorescence collected with appropriate band-pass filters using the following laser-filter combinations: 640 nm diode laser (either iBeam Smart, Toptica, Germany, or Cube 640-100C, Coherent, USA) and 676/37 or 679/41 (Semrock, USA) for AF647, 561/568 nm laser (either 561 DPSS, Oxxius, France, or 568LP, Coherent) and 607/70 (Semrock) for AF568, 532 nm Nd:YAG laser (Nano 250-532-100, Linos, Germany) and 582/75 (Semrock) for ATTO532, 491 nm DPSS laser (Cobolt, Sweden) and 530/55 (Semrock) for EYFP and 405 nm diode laser with 530/55 (Semrock) for mTurquoise. For single-colour *d*STORM imaging using AF647, the switching buffer was composed of 100 mM mercaptoethylamine (Sigma) in PBS solution at pH 8.2. For each field of view, a time series of up to 30,000 frames was acquired with 15 ms exposure and 1–2 kW cm^−2^ irradiance. Subsequently, fluorescence emission from AF568 and mTurquoise were acquired (100 frames and 100 ms exposure).

For two-colour *d*STORM acquisition, between 15,000 and 30,000 frames were acquired for each channel at a frame rate of 10–100 Hz. AF647/AF568, AF647/AF546 and AF647/ATTO532 were excited with irradiation intensities at 2–5 kW cm^−2^ in switching buffer (Supplementary Note 7). The imaging of purified viruses was carried out using TIRF illumination. For cell imaging, highly inclined illumination^67^ was used.

### Localization and super-resolution image reconstruction

All *d*STORM data set were analysed using rapidSTORM 3.3 (ref. 68). In addition, for two-colour *d*STORM imaging, the chromatic offset between the channels was corrected by using image registration (Fiji, bUnwarpJ^69^). The chromatic transformations were obtained from white fluorescent microspheres (100 nm TetraSpeck microsphere, Invitrogen).

### Virus particle averaging and parameter fitting analysis

All analysis subsequent to *d*STORM image reconstruction was performed using custom-written MATLAB routines (Mathworks). Briefly, the fluorescence image stacks obtained for mTurquoise and AF568 were independently averaged and converted into masks by thresholding. The masks obtained were applied to the *d*STORM image. The protein labelled with AF568 was chosen such that it is located in a different structure than that labelled with AF647. When AF647 was used to label a protein present in the tegument, AF568 was used to label a protein in the envelope (glycoproteins). Inversely, if AF647 was used to label a protein from the envelope, AF568 was used to label a protein in the tegument. Virus particles present in the masked *d*STORM image were then located by circular Hough transform^70^, allowing detection of circular structures and determination of the coordinates of their centre (see Supplementary Note 1).

The coordinates of the centre of each particle identified by circular Hough transform were used for particle alignment. The aligned localizations were used to produce the high-density super-resolved image, using the same image reconstruction as that used by rapidSTORM. The diameter and thickness of each protein layer was obtained by weighted non-linear least-square fitting (Levenberg–Marquardt algorithm) of the radial distribution from the aligned localization coordinates to that of the MCV model.

The goodness of fit was assessed by calculating the corresponding reduced *χ*^*2*^ parameter as shown in equation (1).

Where *N(r*_i_) is the value of the experimental histogram at the radius bin *r*_i_, *N*_m_*(r*_i_) is the value of the histogram at the radius *r*_i_ obtained from the MCV model, *n* is the total number of bins in the radius histogram and *p* is the number of fitted parameters (*p*=2).

The two fitted parameters (the shell diameter and thickness) are determined from the optimal fit along with their 95% confidence interval. In some cases, the analysis returned a value of zero for the thickness parameter. The thickness was then considered not determinable (shown as n.d. in Fig. 3).

The analysis of the capsid spatial offset is described in details in Supplementary Note 6.

The MATLAB routines for model-based and capsid spatial offset analysis used in this study are available from our website: http://wiki.laser.ceb.cam.ac.uk/wiki/index.php/Resources.

## Additional information

**How to cite this article**: Laine, R. F. *et al.* Structural analysis of herpes simplex virus by optical super-resolution imaging. *Nat. Commun.* 6:5980 doi: 10.1038/ncomms6980 (2015).

## Supplementary information

Supplementary Information Supplementary Figures 1-5, Supplementary Notes 1-7, and Supplementary References (PDF 744 kb)

## References

[1] Farooq AV, Shukla D (2012). Herpes simplex epithelial and stromal keratitis: an epidemiologic update. Surv. Ophthalmol..

[2] Whitley RJ, Kimberlin DW (2005). Herpes simplex encephalitis: children and adolescents. Semin. Pediatr. Infect. Dis..

[3] Nicoll MP, Proença JT, Efstathiou S (2012). The molecular basis of herpes simplex virus latency. FEMS Microbiol. Rev..

[4] Grünewald K (2003). Three-dimensional structure of herpes simplex virus from cryo-electron tomography. Science.

[5] Zhou ZH (2000). Seeing the herpesvirus capsid at 8.5 Å. Science.

[6] Brown JC, Newcomb WW (2011). Herpesvirus capsid assembly: insights from structural analysis. Curr. Opin. Virol..

[7] Heldwein EE, Krummenacher C (2008). Entry of herpesviruses into mammalian cells. Cell. Mol. Life Sci..

[8] Roizman, H. & Knipe, D. in*Fields Virology* 2399–2460Lippincott Williams and Wilkins (2001).

[9] Desai PJ (2000). A null mutation in the UL36 gene of herpes simplex virus type 1 results in accumulation of unenveloped DNA-filled capsids in the cytoplasm of infected cells. J. Virol..

[10] Roberts APE (2009). Differing roles of inner tegument proteins pUL36 and pUL37 during entry of herpes simplex virus type 1. J. Virol..

[11] Bucks MA, Murphy MA, O’Regan KJ, Courtney RJ (2011). Identification of interaction domains within the UL37 tegument protein of herpes simplex virus type 1. Virology.

[12] Vittone V (2005). Determination of interactions between tegument proteins of herpes simplex virus type 1. J. Virol..

[13] Sandbaumhüter M (2013). Cytosolic herpes simplex virus capsids not only require binding inner tegument protein pUL36 but also pUL37 for active transport prior to secondary envelopment. Cell. Microbiol..

[14] Svobodova S, Bell S, Crump CM (2012). Analysis of the interaction between the essential herpes simplex virus 1 tegument proteins VP16 and VP1/2. J. Virol..

[15] Mossman KL, Sherburne R, Lavery C, Duncan J, Smiley JR (2000). Evidence that herpes simplex virus VP16 is required for viral egress downstream of the initial envelopment event. J. Virol..

[16] Hell SW (2009). Microscopy and its focal switch. Nat. Methods.

[17] Patterson G, Davidson M, Manley S, Lippincott-Schwartz J (2010). Superresolution imaging using single-molecule localization. Annu. Rev. Phys. Chem..

[18] van de Linde S, Heilemann M, Sauer M (2012). Live-cell super-resolution imaging with synthetic fluorophores. Annu. Rev. Phys. Chem..

[19] Hell SW, Wichmann J (1994). Breaking the diffraction resolution limit by stimulated emission: stimulated-emission-depletion fluorescence microscopy. Opt. Lett..

[20] Betzig E (2006). Imaging intracellular fluorescent proteins at nanometer resolution. Science.

[21] Rust M, Bates M, Zhuang X (2006). Sub-diffraction-limit imaging by stochastic optical reconstruction microscopy (STORM). Nat. Methods.

[22] van de Linde S (2011). Direct stochastic optical reconstruction microscopy with standard fluorescent probes. Nat. Protoc..

[23] Ivanchenko S (2009). Dynamics of HIV-1 assembly and release. PLoS Pathog..

[24] Chojnacki J (2012). Maturation-dependent HIV-1 surface protein redistribution revealed by fluorescence nanoscopy. Science.

[25] He J (2013). Dual function of CD81 in influenza virus uncoating and budding. PLoS Pathog..

[26] Malkusch S, Muranyi W, Müller B, Kräusslich H-G, Heilemann M (2013). Single-molecule coordinate-based analysis of the morphology of HIV-1 assembly sites with near-molecular spatial resolution. Histochem. Cell Biol..

[27] Muranyi W, Malkusch S, Müller B, Heilemann M, Kräusslich H-G (2013). Super-resolution microscopy reveals specific recruitment of HIV-1 envelope proteins to viral assembly sites dependent on the envelope C-terminal tail. PLoS Pathog..

[28] Annibale P, Vanni S, Scarselli M, Rothlisberger U, Radenovic A (2011). Quantitative photo activated localization microscopy: unraveling the effects of photoblinking. PLoS ONE.

[29] Lando D (2012). Quantitative single-molecule microscopy reveals that CENP-A(Cnp1) deposition occurs during G2 in fission yeast. Open Biol..

[30] Ehmann N (2014). Quantitative super-resolution imaging of Bruchpilot distinguishes active zone states. Nat. Commun..

[31] Hess ST (2007). Dynamic clustered distribution of hemagglutinin resolved at 40 nm in living cell membranes discriminates between raft theories. Proc. Natl Acad. Sci. USA.

[32] Sengupta P (2011). Probing protein heterogeneity in the plasma membrane using PALM and pair correlation analysis. Nat. Methods.

[33] Schmied JJ (2014). DNA origami-based standards for quantitative fluorescence microscopy. Nat. Protoc..

[34] Löschberger A (2012). Super-resolution imaging visualizes the eightfold symmetry of gp210 proteins around the nuclear pore complex and resolves the central channel with nanometer resolution. J. Cell Sci..

[35] Crump CM (2004). Alphaherpesvirus glycoprotein M causes the relocalization of plasma membrane proteins. J. Gen. Virol..

[36] Heilemann M (2008). Subdiffraction-resolution fluorescence imaging with conventional fluorescent probes. Angew. Chem. Int. Ed. Engl..

[37] Deschout H (2014). Precisely and accurately localizing single emitters in fluorescence microscopy. Nat. Methods.

[38] Mortensen KI, Churchman LS, Spudich JA, Flyvbjerg H (2010). Optimized localization analysis for single-molecule tracking and super-resolution microscopy. Nat. Methods.

[39] Harris LJ, Larson SB, Hasel KW, McPherson A (1997). Refined structure of an intact IgG2a monoclonal antibody. Biochemistry.

[40] Schrag JD, Prasad BVV, Rixon FJ, Chiu W (1989). Three-dimensional structure of the HSV1 nucleocapsid. Cell.

[41] Zhu Q, Courtney RJ (1994). Chemical cross-linking of virion envelope and tegument proteins of herpes simplex virus type 1. Virology.

[42] Gross ST, Harley CA, Wilson DW (2003). The cytoplasmic tail of Herpes simplex virus glycoprotein H binds to the tegument protein VP16 in vitro and in vivo. Virology.

[43] Coller KE, Lee JI-H, Ueda A, Smith GA (2007). The capsid and tegument of the alphaherpesviruses are linked by an interaction between the UL25 and VP1/2 proteins. J. Virol..

[44] Mijatov B, Cunningham AL, Diefenbach RJ (2007). Residues F593 and E596 of HSV-1 tegument protein pUL36 (VP1/2) mediate binding of tegument protein pUL37. Virology.

[45] Schmid MF (2012). A tail-like assembly at the portal vertex in intact herpes simplex type-1 virions. PLoS Pathog..

[46] Bohannon KP, Jun Y, Gross SP, Smith GA (2013). Differential protein partitioning within the herpesvirus tegument and envelope underlies a complex and variable virion architecture. Proc. Natl Acad. Sci. USA.

[47] Szymborska A (2013). Nuclear pore scaffold structure analyzed by super-resolution microscopy and particle averaging. Science.

[48] Ries J, Kaplan C, Platonova E, Eghlidi H, Ewers H (2012). A simple, versatile method for GFP-based super-resolution microscopy via nanobodies. Nat. Methods.

[49] Opazo F (2012). Aptamers as potential tools for super-resolution microscopy. Nat. Methods.

[50] Lew MD, Backlund MP, Moerner WE (2013). Rotational mobility of single molecules affects localization accuracy in super-resolution fluorescence microscopy. Nano Lett..

[51] Newcomb WW, Brown JC (2010). Structure and capsid association of the herpesvirus large tegument protein UL36. J. Virol..

[52] Jambunathan N (2014). Herpes simplex virus 1 protein UL37 interacts with viral glycoprotein gK and membrane protein UL20 and functions in cytoplasmic virion envelopment. J. Virol..

[53] Newcomb WW, Jones LM, Dee A, Chaudhry F, Brown JC (2012). Role of a reducing environment in disassembly of the herpesvirus tegument. Virology.

[54] Kelly BJ, Fraefel C, Cunningham AL, Diefenbach RJ (2009). Functional roles of the tegument proteins of herpes simplex virus type 1. Virus Res..

[55] Huang B, Wang W, Bates M, Zhuang X (2008). Three-dimensional super-resolution imaging by stochastic optical reconstruction microscopy. Science.

[56] Juette MF (2008). Three-dimensional sub-100 nm resolution fluorescence microscopy of thick samples. Nat. Methods.

[57] Löschberger A, Franke C, Krohne G, van de Linde S, Sauer M (2014). Correlative super-resolution fluorescence and electron microscopy of the nuclear pore complex with molecular resolution. J. Cell Sci..

[58] Boukamp P (1988). Normal keratinization in a spontaneously immortalized aneuploid human keratinocyte cell line. J. Cell Biol..

[59] Sharma GG (2003). hTERT associates with human telomeres and enhances genomic stability and DNA repair. Oncogene.

[60] Gierasch WW (2006). Construction and characterization of bacterial artificial chromosomes containing HSV-1 strains 17 and KOS. J. Virol. Methods.

[61] Tischer BK, Smith GA, Osterrieder N (2010). En passant mutagenesis: a two step markerless red recombination system. Methods Mol. Biol..

[62] McLean C (1982). Monoclonal antibodies to three non-glycosylated antigens of herpes simplex virus type 2. J. Gen. Virol..

[63] Minson AC (1986). An analysis of the biological properties of monoclonal antibodies against glycoprotein D of herpes simplex virus and identification of amino acid substitutions that confer resistance to neutralization. J. Gen. Virol..

[64] Gill MB (2006). Murine gammaherpesvirus-68 glycoprotein H-glycoprotein L complex is a major target for neutralizing monoclonal antibodies. J. Gen. Virol..

[65] Erdelyi M (2013). Correcting chromatic offset in multicolor super-resolution localization microscopy. Opt. Express.

[66] Kaminski Schierle GS (2011). In situ measurements of the formation and morphology of intracellular β-amyloid fibrils by super-resolution fluorescence imaging. J. Am. Chem. Soc..

[67] Tokunaga M, Imamoto N, Sakata-Sogawa K (2008). Highly inclined thin illumination enables clear single-molecule imaging in cells. Nat. Methods.

[68] Wolter S (2012). rapidSTORM: accurate, fast open-source software for localization microscopy. Nat. Methods.

[69] Arganda-Carreras I (2006). Computer Vision Approaches to Medical Image Analysis.

[70] Atherton TJ, Kerbyson DJ (1999). Size invariant circle detection. Image Vis. Comput..

