# Supplementary material for: Structural analysis of herpes simplex virus by optical super-resolution imaging
Source: Nat Commun. 2015 Jan 22;6:5980. doi: 10.1038/ncomms6980 (PMC4338551; doi:10.1038/ncomms6980)
Supplement: Supplementary Information — Supplementary Figures 1-5, Supplementary Notes 1-7, and Supplementary References (PDF 744 kb) [file 41467_2015_BFncomms6980_MOESM252_ESM.pdf]

# Structural analysis of Herpes Simplex Virus by optical super-resolution imaging

## Supplementary information

Romain F. Laine[1#], Anna Albecka[2#], Sebastian van de Linde[3], Eric J. Rees[1], Colin M. Crump[2*], Clemens F. Kaminski[1*]

1. Laser Analytics Group, Department of Chemical Engineering and Biotechnology, Cambridge University, Cambridge, UK.
2. Division of Virology, Department of Pathology, Cambridge University, Cambridge, UK.
3. Department of Biotechnology & Biophysics, Julius-Maximilians-University, Würzburg, Germany

[#] These authors contributed equally to this work.
* Corresponding authors: Prof. Clemens Kaminski cfk23@cam.ac.uk and Dr. Colin Crump cmc56@cam.ac.uk

## Supplementary figures

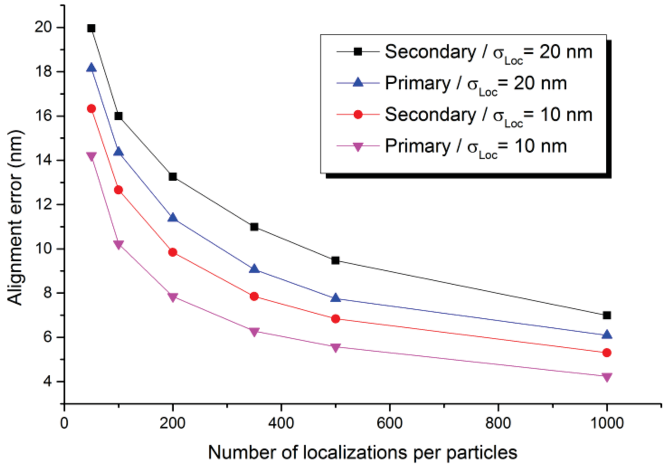


**Supplementary Figure 1. Error on determining the centre of the particle using the cHough.** The error on the determination of the centre of individual particles (alignment particle) using the cHough method was estimated by simulation (see Methods section for details). The error is defined here as the standard deviation of the error. Here, a diameter of 80 nm and a thickness of 30 nm were used. Several combinations of linker size (secondary and primary labelling) and localization error ($\sigma_{Loc}$) were tested.

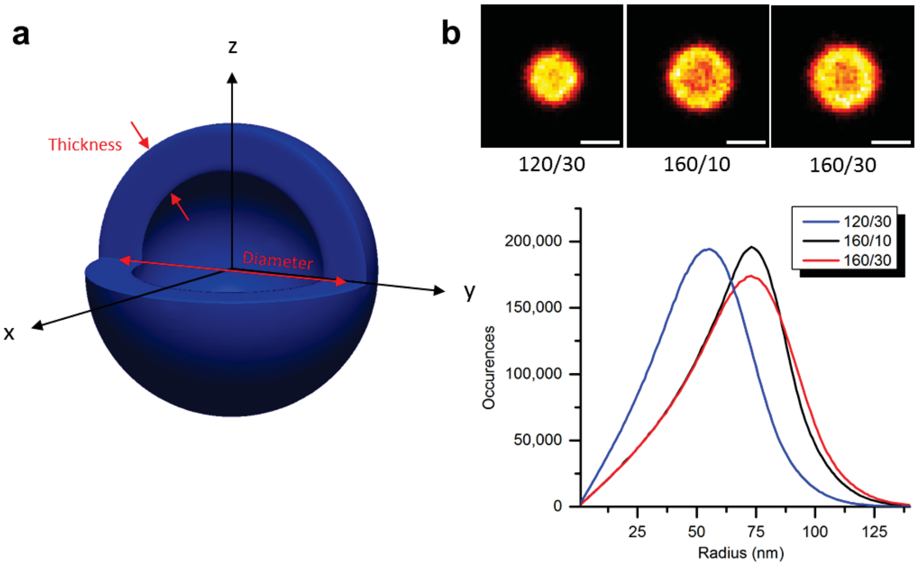


**Supplementary Figure 2. MCV model.** (**a**) Protein shell used by the MCV model. Its descriptive parameters (diameter and thickness) are indicated. (**b**) Virus images and corresponding radial histograms obtained from the MCV model for different values of diameter and thickness. Diameter and thickness are indicated as diameter/thickness (for instance, 120/30 represents a 120 nm diameter and 30 nm thickness). The other simulation parameters were set as follows: 15 nm localization error and primary labelling (10 nm linker size). The virus images were reconstructed from 10,000 localizations and the radial histograms were evaluated from 10,000,000 localizations. Scale bar: 100 nm

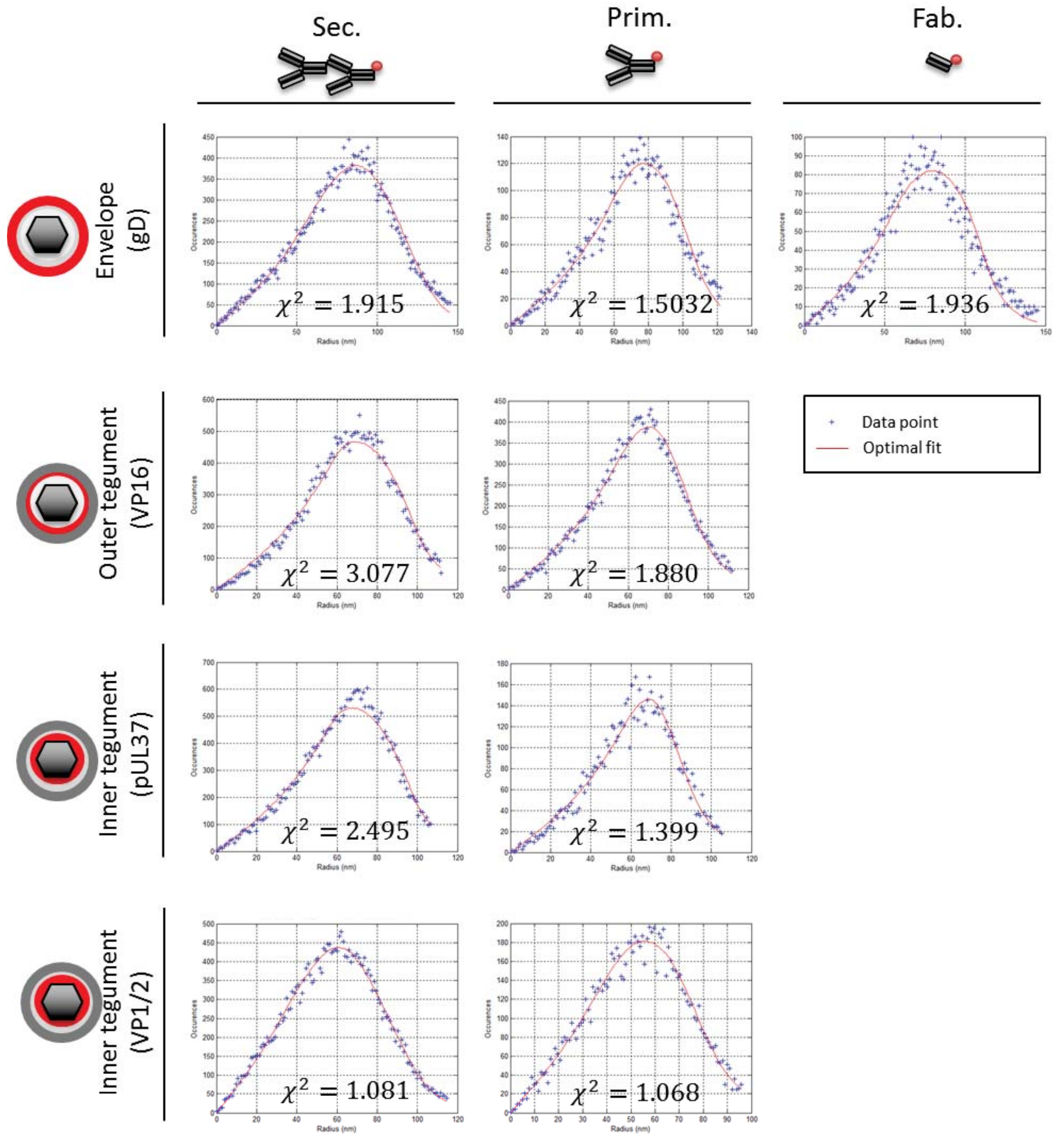


**Supplementary Figure 3. Radial distribution and optimal fit obtained from the model-based analysis.** The optimal fit was obtained by weighted non-linear least square minimisation. The corresponding optimal shell diameter and thickness parameters are shown in Figure 3. The goodness of the fit parameters ($\chi^2$) are also indicated.

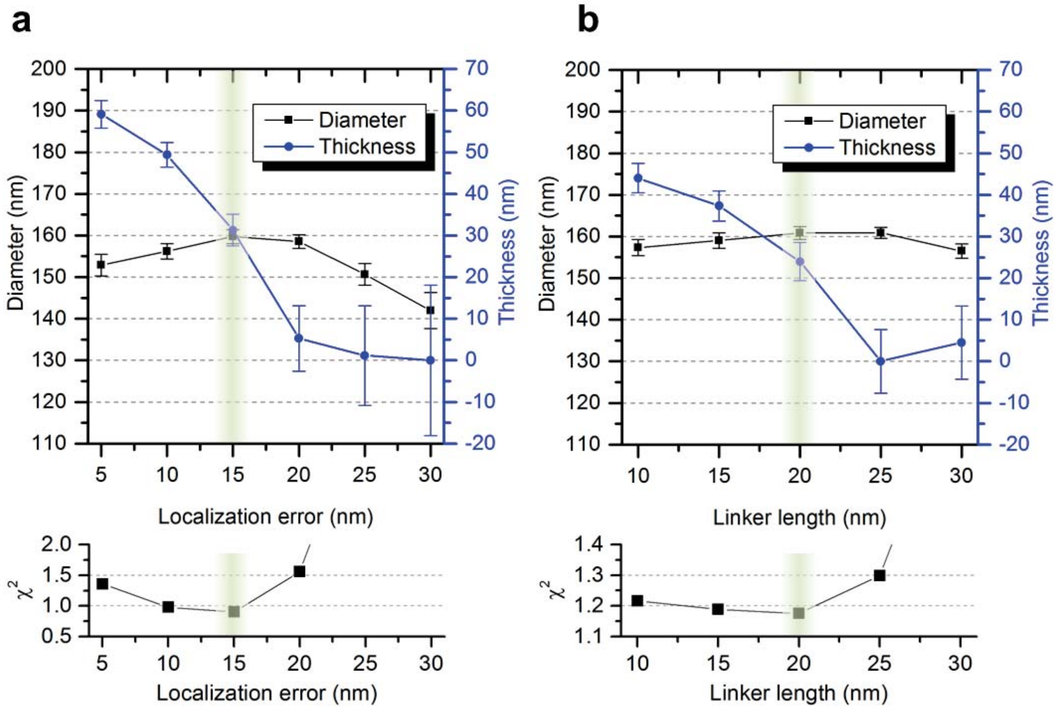


**Supplementary Figure 4. Influence of the linker length and localization error on the fitted parameters.** (**a**) Shell radius and thickness obtained as a result of the fit with varying localization error. (**b**) Shell radius and thickness obtained as a result of the fit with varying linker length. The dataset was simulated with a diameter of 160 nm, thickness of 30 nm, localization error of 15 nm and linker length of 20 nm, with a total number of localizations of 10,000. The quality of the fit can be assessed by the fitting criterion ($\chi^2$). The simulated values are highlighted with the green shading.

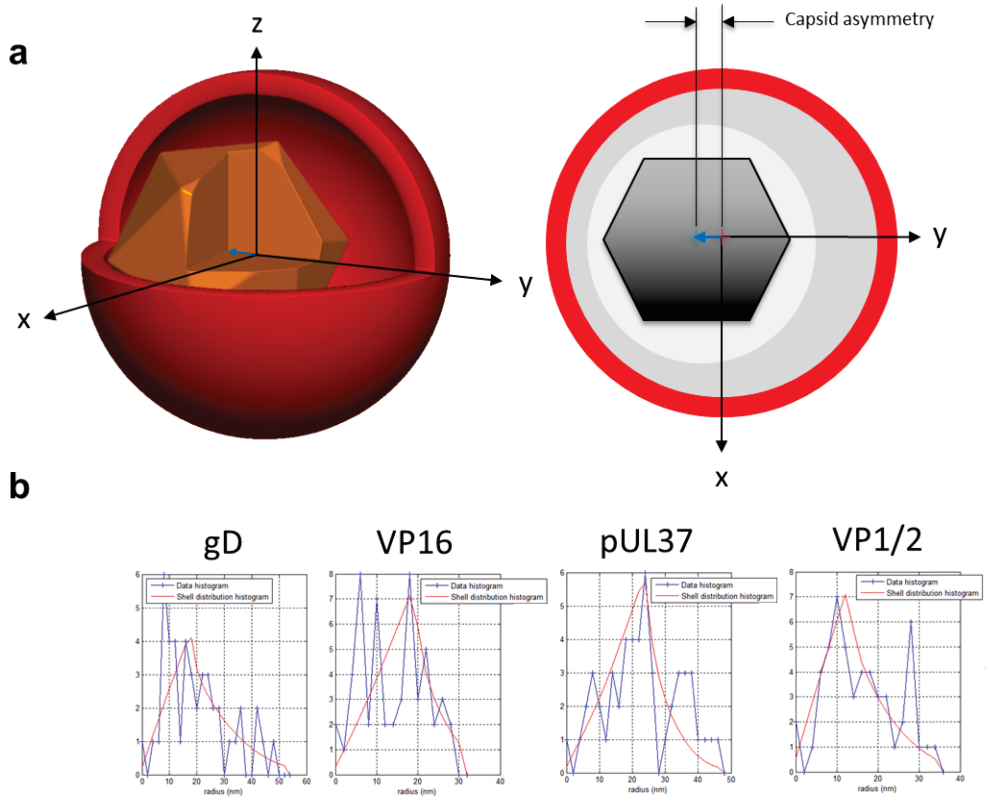


| | | | | |
|---|---|---|---|---|
| Offset (nm) | 19.3 nm (17.4 - 24.9) ($\chi^2$ = 0.748) | 20.3 nm (18.8 – 24.0) ($\chi^2$ = 1.939) | 25.4 nm (23.7 – 26.5) ($\chi^2$ = 1.0474) | 14 nm (12.4 – 19.6) ($\chi^2$ = 0.981) |
| Corrected offset (nm) | 12.0 nm (10.5 - 14.1) ($\chi^2$ = 0.688) | 7.3 nm (5.9 – 8.8) ($\chi^2$ = 1.538) | 24.3 nm (21.7 – 26.2) ($\chi^2$ = 0.771) | NA |
| Particles | 45 | 54 | 52 | 54 |

**Supplementary Figure 5. Analysis of the capsid spatial offset.** (**a**) Diagrams showing the spatial offset between the centre of the capsid and that of the outer protein layers. (**b**) Results of the capsid offset analysis. The radial distribution and the optimal fit are shown. The optimal values of the offset and corrected offset are shown in the table. The values in brackets are the lower and upper bound obtained from the 95% confidence interval of the exhaustive search fitting.

## Supplementary data

**Supplementary note 1. Determining the centre of each virus particles using circular Hough transform.**

The precise coordinates of the centre of each virus particle were determined by applying a circle detection algorithm based on the circular Hough transform[1] (cHough). To the reconstructed super-resolution image (in which circular viruses can be readily observed, see Figure 2a and 3), the determination of the centre of each particle is essential for the particle alignment procedure. The use of the cHough algorithm for the detection of the virus and the determination of their centre is a more robust and more efficient approach than object detection followed by calculation of the centre of mass. First, because the detection and the determination of the centre are determined simultaneously and not in two separate analysis steps but, also, because the circle detection of the cHough is very robust to occlusion, particle clusters, non-uniform labelling and presence of background.

The error on the determination of the centre of an individual particle by the cHough method can be estimated by simulation using the MCV model. For that, we simulated 10,000 particles using parameters similar to those obtained experimentally. Importantly, the same number of localizations per particles as experimentally obtained was used (typically 100-300 localizations per particle, see Figure 3 for details). Those simulated particles were analysed by cHough using the same routine as with the experimental data. The distribution of error between the estimated and simulated centre of particle was obtained and found to be approximatively Gaussian. This standard deviation of the error distribution was used as the estimator of the error on the determination of the centre of an individual particle (further called alignment error).

Supplementary Fig. 1 shows that the variation of the alignment error as a function of the number of localization per particle. Here, the use of primary or secondary labelling with either 10 or 20 nm average localization precision was tested. We note that, as the number of localization increases the error decreases. This is in agreement with the fact that more localization will render a better defined particle, for which the centre can be determined with higher accuracy. For a fixed number of localizations per particle, decreasing the labelling size or the localization error both result in a lower alignment error. In fact, both contribute to a more compact particle for which the edges and therefore the centre can be determined with higher precision. However, secondary labelling often exhibited a larger number of localization per particle (see Figure 3), therefore compensating for the larger linker size. Therefore, the alignment error was relatively constant across dataset (~10 nm), of the order of the localization error.

This alignment error was taken into account in the analysis by introducing the total localization error as the quadratic sum of both the average single-molecule localization error and the alignment error as shown in equation (2).

**Supplementary note 2. Monte-Carlo virus model (MCV model).**

The MCV model is described in Figure 2b. Briefly, the model is based on Monte-Carlo simulations of localization data obtained from proteins randomly distributed in a spherical shell (described by its diameter and its thickness, as shown in Supplementary Fig. 2a), displaced by the linker size (effect of labelling) and affected by localization error.

First, a large number of protein coordinates (10,000,000) are generated by random point-picking within a 3D spherical shell with a fixed radius and thickness (Supplementary Fig. 2a). For each protein position, the displacement induced by the linker size is simulated by adding a spatial offset randomly drawn from another shell point-picking with radius and thickness corresponding to the type of labelling. The labelling linker size was estimated from the structure of the IgG (PDB 1IGT[2]). Unless specifically notified, linker sizes were set as follows: 20 nm ± 2.5 nm for secondary labelling, 10 nm ± 1 nm for primary and 5 nm ± 1 nm for Fab fragments. The flexibility of the linker is introduced by the thickness of the shell used to generate the linker displacement.

The 3D coordinates are then projected onto the 2D imaging plane to simulate the 2D dSTORM imaging. In order to take the localization error into account, each 2D coordinates were then further displaced by an offset picked from a 2D Gaussian distribution with standard deviation corresponding to the total localization error.

The total localization error can be estimated from the single-molecule localization error and the particle alignment error (see Supplementary Data 3 for details). From this set of coordinates simulated by the MCV model, the super-resolved image of the virus can be reconstructed and the radial distribution of localization data can be evaluated. In Supplementary Fig. 2b, three different dataset were generated with different structural parameters (120/30 for 120 nm diameter and 30 nm thickness, 160/10 for 160 nm diameter and 10 nm thickness and 160/30 for 160 nm diameter and 30 nm thickness). We note that the virus images appeared very similar to those shown in Figure 3. Additionally, the radial histograms shown here indicate the effect of the change in diameter or thickness on the shape of the distribution. A reduction in the diameter (160/30 to 120/30) resulted in a shift of the distribution to shorter radii, whereas a reduction in the thickness (160/30 to 160/10) resulted in a sharpening of the distribution.

In the model-based analysis, the model parameters can be iteratively fitted to determine the protein shell diameter and thickness that best describes the experimental radial distribution.

**Supplementary note 3. Estimation of the total localization precision.**

The MCV model is based on the assumption that a particular viral protein is distributed in a shell with spherical symmetry (as shown in Figure 2b). The spatial distribution of the protein is described by the diameter and thickness of the protein shell. Those two parameters are fitted in the model-based analysis. There are two further parameters in the model: the linker length and the total localization error. Those two supplementary parameters are estimated and fixed during the analysis. The linker length is estimated as described in Supplementary Data 2.

In the aligned localization dataset, two main sources of error affect the localizations: the single-molecule localization precision and the error introduced when aligning the particles. The latter corresponds to the error in determining the coordinates of the centre of an individual particle by cHough (alignment error). Provided a large number of particles are considered, the total localization error can be obtained from the quadratic sum of the average localization error (single-molecule localization precision) and the alignment error, as showed in equation (2).

$$\sigma_{Tot}^2 = \sigma_{Loc}^2 + \sigma_{cHough}^2 \qquad (2)$$

The localization error ($\sigma_{Loc}$) was estimated using the formulae for the standard deviation of the localization estimator presented by Mortensen et al.[3]. For this, the background (5-15 photons) and the full-width at half maximum of the point spread function size (~340 nm) were estimated from single frames of typical experimental SMLM dataset acquired using purified viruses labelled with AF647. The pixel size corresponding to the imaging set-up was used (160 nm).

From the distribution of localization precision obtained from the aligned localizations, the mean localization precision was calculated and used as $\sigma_{Loc}$. Similarly, for each dataset, the alignment error ($\sigma_{cHough}$) was estimated by simulation (see Supplementary Data 1 for details), using parameters describing the experimental dataset as closely as possible (same number of localizations per particles, linker parameters, localization precision and protein shell parameters). Typically, both the localization error and the alignment error were of the order of 10 nm, leading therefore to a total localization error of the order of 15 nm.

**Supplementary note 4. Robustness of the estimation of the shell diameter.**

We were also interested in testing the robustness of our model-based analysis. For this, we generated representative aligned localization data using the MCV model (80 nm radius, 30 nm thickness, 15 nm localization error and 20 nm linker length with a total number of localizations of 10,000) and carried out a series of fits with varying localization precision. As shown in Supplementary Fig. 4a, an error on the localization error of almost 70% was necessary to see a deviation of the returned radius of 5% compared to the true value.

Using a similar method, the effect of the linker length was investigated (see Supplementary Fig. 4b). Here, an error of 50% on the linker length led to a deviation from the true value of the radius of less than 2%. We conclude that the radius returned by our analysis is very robust with respect to the fixed parameters (linker parameters and localization error). Importantly, we also confirmed that the quality of the fit, assessed by the fitting criterion $\chi^2$ and described in equation (1), was minimised when the correct value of localization error and linker length were used.

**Supplementary note 5. Variability of the linker length with secondary antibody labelling.**

Previously, we used the MCV model to fit the virus protein shell diameter and thickness, and the linker size and localization precision were takin as fixed parameters based on the structure of the IgG and the localization error estimator provided by Mortensen et al., respectively. Since the localization error can be estimated by a method based on experimental data (number of photons), we think that biases are more likely to occur from an erroneous estimation of the linker size rather than an erroneous estimation of the localization error.

In fact, it is likely that where the analysis was unable to return a value (not determinable in Figure 3), an error in the estimation of the linker size may have occurred. Here, we then investigated adjusting the linker size for the corresponding experimental dataset (VP16 and pUL37).

For both VP16 and pUL37 with secondary labelling, the quality of the fit could be significantly improved by allowing the linker size to vary. We obtained an optimum linker size of 15 nm for both proteins shown by a significant decrease in the $\chi^2$ parameter, from 3.077 to 2.586 for VP16 and from 2.495 to 1.470 for pUL37. The recovered diameter were 160.0 ± 1.4 nm and 157.8 ± 0.8 nm for VP16 and pUL37 respectively. Those values are in good agreement with the values shown in Figure 3, in accordance with the robustness of the shell diameter estimation as shown in Supplementary Data 4. The values of thickness obtained after adjustment of the linker size here were 19 ± 4 nm and 11 ± 4 nm for VP16 and pUL37 respectively. Those values also appear in good agreement with those obtained from the primary labelling.

For all dataset obtained using primary labelling, the optimal fit was obtained for the expected linker size of 10 nm. Increasing or decreasing the linker size only decreased the quality of the fit (increase in $\chi^2$). This supports the fact that the estimation of linker size of 10 nm is a good representation of the true structure of the labelling in our experimental dataset.

We can then conclude that the secondary labelling appear more compact than the standard estimated linker length of 20 nm in the case of those two proteins, and a linker size of 15 nm describes the dataset better than the 20 nm. This is consistent with the notion that those proteins are buried into the tegument shell which is likely to affect the structure of the labelling, compared to the unconstrained structure.

Similarly, the fit of the dataset obtained from gD with secondary labelling was improved by adjusting the linker length to 25 nm ($\chi^2$ decreased from 1.915 to 1.794). In this case, the diameter and thickness recovered were 191.0 ± 1.4 nm and 44 ± 3 nm respectively. Here again, the fitted diameter is unaffected by the change of linker size and the thickness is now in better agreement with that obtained from the primary labelling (40 ± 5 nm, see Figure 3). This observation implies that, in this case, the secondary labelling appears longer than expected and suggests that the linker is instead stretched out since a linker size of 25 nm describes the dataset better. This is also in agreement with the idea that gD is an envelope protein and that the antibodies are likely to be pointing outwards from the membrane.

Therefore, the linker size parameter appears largely variable in the case of secondary labelling and significantly influences the output of our analysis for the thickness parameter. In contrast, the values obtained for diameter and thickness with primary labelling appear robust and reliable with a single linker size of 10 nm.

In conclusion, this analysis suggests that the dataset obtained from secondary antibodies are more subject to bias, notably from erroneous estimation of the linker length (due to its large variability depending on the protein labelled) and from an aggravating effect when a deviation from the spherical geometry is present (for instance with non-random labelling such as labelling preferentially pointing outwards as in the case of the envelope protein gD). Here we observed that, the linker length for secondary labelling could vary between 15 and 25 nm depending on the type of protein that is labelled, whereas primary labelling was consistently well described with a linker size of 10 nm.

**Supplementary note 6. Estimation of the capsid spatial offset and model.**

The capsid offset analysis was performed using a custom-written MATLAB routine. For each individual particle in a field of view, the centre of the protein layer (envelope or tegument) was determined from the dSTORM image using the cHough. The centre of the capsid was precisely determined by localization of the fluorescence ensemble from mTurquoise-VP26 using rapidSTORM with a full width half maximum of the point spread function of 500 nm. The mTurquoise localizations were corrected for chromatic aberration with a two-colour transformation using rapidSTORM. The transformation between the two channels was obtained with the method described for 2-colour *d*STORM. Additionally, a linear spatial offset affected the localizations obtained between the two channels. This linear offset was constant across a single field of view and could therefore be corrected by calculating the centre of mass of each group of data point obtained for each field of view.

By calculating the displacement between the centre of the particle and that of the capsid for numerous particles, the distribution of these distances can be obtained (radial distribution). This distribution was then fitted to our capsid offset model by weighted least-square minimisation using the exhaustive search approach, minimising the fitting criterion as described by equation (1).

Our capsid offset model can be described as follows: for a single particle, the relative position of the centre of the capsid can be located anywhere on a sphere centred on the centre of the outer layer, depending on its orientation. The radius of that sphere represents the spatial offset of the capsid. A schematic representation of the spatial offset is shown in Supplementary Fig. 5a. Our data can be modelled as a set of randomly oriented coordinates on a sphere (random sphere point picking[4]), that is projected on the 2D imaging plane and then affected by a localization error. Similarly to the MCV model, these data can be modelled using Monte-Carlo simulation. In this case, the total localization error is a result of the localization error of the centre of the capsid and the alignment error. The capsid centroid localization error can be estimated using Mortensen et al. formulae and leads to a typical localization error of the order of 5 nm thanks to the high signal to noise ratio obtained in the mTurquoise dataset. The alignment error represents the error on the determination of the coordinates of the centre of the particle and can be estimated as described in Supplementary Data 1.

The results of the capsid offset analysis are shown in in Supplementary Fig. 5b. We observe an offset of ~20 nm for both gD and VP16, a slightly larger estimation compared to previous measurement using ET[5]. An even higher offset was measured for pUL37 (~25 nm). For VP1/2, an offset of 14 nm was measured. This lower offset value may reflect the fact that this capsid-bound protein is thought to be located close to the capsid and is expected to exhibit little to no offset[6]. This value of offset may then be a result of an analysis bias due to the fact that we underestimated the total localization precision. In fact, as we showed previously[7], it is difficult to totally correct for the chromatic offset and random residual error may still reside. It is reasonable to think that the 14 nm offset observed may then correspond to this uncorrected residual random error.

Therefore, if we assume that an extra 14 nm random error contributes on all dataset, the offset obtained for the other proteins (gD, VP16 and pUL37) can be analysed again taking into account the additional localization error (assuming that the quadratic summation of errors is valid). The corrected offsets obtained this way appear shorter than the uncorrected ones. Furthermore, the assumption of the extra random error due to chromatic offset is supported by the improvement on the quality of the fit observed when using the additional localization error (decrease of the $\chi^2$ parameter, as shown in Supplementary Fig. 5b).

**Supplementary note 7: Imaging buffers**

Since photoswitching properties can vary within different biological samples, the optimal conditions for imaging must be obtained by testing. For all *d*STORM acquisition presented here, the switching buffer was composed of 100 mM MEA in PBS at various pH depending on the dye combination. As previously shown[8], MEA acts already as an oxygen scavenger at higher concentration (e.g., 100 mM).. A combination of 50 mM MEA and additional enzymatic oxygen scavenging system as previously described[9] was also tested, but here it led to a worse photoswitching properties than with 100 mM MEA only (notably fast photoswitching rates leading to local high densities incompatible with our imaging capabilities).

For single-colour *d*STORM imaging using AF647, the optimal pH was found at pH 8.2. In combination with AF568, a buffer pH compatible with both dyes simultaneously was necessary and the pH had to be slightly increased to 8.7 in order to improve the photoswitching properties of AF568. Similarly, the pH was adjusted for the other dye combinations as follows: pH 7.2 for AF647 and AF546, and pH 8.0 – 9.0 for AF647 and ATTO532. In all cases, the photoswitching properties of AF647 were not significantly affected by that pH change.

## References


1. Atherton, T. J. & Kerbyson, D. J. Size invariant circle detection. *Image Vis. Comput.* **17,** 795–803 (1999).

2. Harris, L. J., Larson, S. B., Hasel, K. W. & McPherson, A. Refined structure of an intact IgG2a monoclonal antibody. *Biochemistry* **36,** 1581–97 (1997).

3. Mortensen, K. I., Churchman, L. S., Spudich, J. A. & Flyvbjerg, H. Optimized localization analysis for single-molecule tracking and super-resolution microscopy. *Nat. Methods* **7,** 377–81 (2010).

4. Weisstein, E. W. Sphere Point Picking. *MathWorld* at http://mathworld.wolfram.com/SpherePointPicking.html on September 18, 2014

5. Grünewald, K. *et al.* Three-dimensional structure of herpes simplex virus from cryo-electron tomography. *Science* **302,** 1396–8 (2003).

6. Bohannon, K. P., Jun, Y., Gross, S. P. & Smith, G. A. Differential protein partitioning within the herpesvirus tegument and envelope underlies a complex and variable virion architecture. *Proc. Natl. Acad. Sci. U. S. A.* **110,** E1613–20 (2013).

7. Erdelyi, M. *et al.* Correcting chromatic offset in multicolor super-resolution localization microscopy. *Opt. Express* **21,** 10978–88 (2013).

8. Schäfer, P., van de Linde, S., Lehmann, J., Sauer, M. & Doose, S. Methylene blue- and thiol-based oxygen depletion for super-resolution imaging. *Anal. Chem.* **85,** 3393–400 (2013).

9. Heilemann, M. *et al.* Subdiffraction-resolution fluorescence imaging with conventional fluorescent probes. *Angew. Chem. Int. Ed. Engl.* **47,** 6172–6 (2008).
